# Multiomics and blood-based biomarkers of moyamoya disease: protocol of Moyamoya Omics Atlas (MOYAOMICS)

**DOI:** 10.1186/s41016-024-00358-3

**Published:** 2024-02-08

**Authors:** Peicong Ge, Zihan Yin, Chuming Tao, Chaofan Zeng, Xiaofan Yu, Shixiong Lei, Junsheng Li, Yuanren Zhai, Long Ma, Qiheng He, Chenglong Liu, Wei Liu, Bojian Zhang, Zhiyao Zheng, Siqi Mou, Zhikang Zhao, Shuang Wang, Wei Sun, Min Guo, Shuai Zheng, Jia Zhang, Xiaofeng Deng, Xingju Liu, Xun Ye, Qian Zhang, Rong Wang, Yan Zhang, Shaosen Zhang, Chengjun Wang, Ziwen Yang, Nijia Zhang, Mingxing Wu, Jian Sun, Yujia Zhou, Zhiyong Shi, Yonggang Ma, Jianpo Zhou, Shaochen Yu, Jiaxi Li, Junli Lu, Faliang Gao, Wenjing Wang, Yanming Chen, Xingen Zhu, Dong Zhang, Jizong Zhao

**Affiliations:** 1 Department of Neurosurgery, Beijing Tiantan Hospital, Capital Medical University, Beijing, China; 2 China National Clinical Research Center for Neurological Diseases, Beijing, China; 3 Department of Neurosurgery, Beijing Hospital, National Center of Gerontology, Beijing, China; 4 Department of Radiology, Beijing Tiantan Hospital, Beijing, China; 5 Department of Ultrasound, Beijing Tiantan Hospital, Capital Medical University, Beijing, China; 6 Department of Neurology, Beijing Tiantan Hospital, Beijing, China; 7 Department of Neurosurgery, Beijing Friendship Hospital, Capital Medical University, Beijing, China; 8 Department of Neurosurgery, Beijing Chaoyang Hospital, Capital Medical University, Beijing, China; 9 Department of Neurosurgery, Beijing Childrens Hospital, Capital Medical University, Beijing, China; 10 Department of Neurosurgery, The Affiliated Children’s Hospital, Capital Institute of Pediatrics, Beijing, China; 11 Department of Neurosurgery, Beijing Changping District Hospital, Beijing, China; 12 Department of Neurosurgery, Dongfang Hospital, Beijing University of Chinese Medicine, Beijing, China; 13 Department of Neurosurgery, Nanjing Drum Tower Hospital, The Affiliated Hospital of Nanjing University Medical School, Nanjing, China; 14 Department of NeuroInterventional Surgery, Binzhou Medical University Hospital, Binzhou, Shandong China; 15 Department of Neurosurgery, Renji Hospital, Shanghai Jiao Tong University School of Medicine, Shanghai, China; 16 Department of Neurosurgery, The First Affiliated Hospital, Zhejiang University School of Medicine, Hangzhou, China; 17 Department of Neurosurgery, The First Affiliated Hospital of Xi’an Jiaotong University, Shaanxi, Xi’an, China; 18 Department of Neurosurgery, West China Hospital, Sichuan University, Chengdu, China; 19 Department of Neurosurgery, Center for Rehabilitation Medicine, Zhejiang Provincial Peoples Hospital, Affiliated Peoples Hospital, Hangzhou Medical College, Hangzhou, Zhejiang China; 20 Beijing Institute of Hepatology, Beijing YouAn Hospital, Capital Medical University, Beijing, China; 21 Department of Neurosurgery, The Second Affiliated Hospital of Soochow University, Suzhou, China; 22 Department of Neurosurgery, The Second Affiliated Hospital of Nanchang University, Nanchang, China

## Abstract

**Background:**

Moyamoya disease (MMD) is a rare and complex cerebrovascular disorder characterized by the progressive narrowing of the internal carotid arteries and the formation of compensatory collateral vessels. The etiology of MMD remains enigmatic, making diagnosis and management challenging. The MOYAOMICS project was initiated to investigate the molecular underpinnings of MMD and explore potential diagnostic and therapeutic strategies.

**Methods:**

The MOYAOMICS project employs a multidisciplinary approach, integrating various omics technologies, including genomics, transcriptomics, proteomics, and metabolomics, to comprehensively examine the molecular signatures associated with MMD pathogenesis. Additionally, we will investigate the potential influence of gut microbiota and brain-gut peptides on MMD development, assessing their suitability as targets for therapeutic strategies and dietary interventions. Radiomics, a specialized field in medical imaging, is utilized to analyze neuroimaging data for early detection and characterization of MMD-related brain changes. Deep learning algorithms are employed to differentiate MMD from other conditions, automating the diagnostic process. We also employ single-cellomics and mass cytometry to precisely study cellular heterogeneity in peripheral blood samples from MMD patients.

**Conclusions:**

The MOYAOMICS project represents a significant step toward comprehending MMD’s molecular underpinnings. This multidisciplinary approach has the potential to revolutionize early diagnosis, patient stratification, and the development of targeted therapies for MMD. The identification of blood-based biomarkers and the integration of multiple omics data are critical for improving the clinical management of MMD and enhancing patient outcomes for this complex disease.

## Background

Moyamoya disease (MMD), classified as a rare and enigmatic cerebrovascular disorder, manifests with the gradual narrowing and blockage of the internal carotid arteries, as well as the development of collateral blood vessels at the base of the brain [1, 2]. The term “Moyamoya” is derived from the Japanese word for “puff of smoke,” which aptly describes the appearance of abnormal collateral vessels on angiography. Although this disease was first reported in Japan, it is now recognized worldwide. MMD predominantly affects the pediatric population but can also manifest in adults, presenting a significant health challenge due to its potential for devastating strokes, cognitive impairments, and other neurological sequelae [3].

Understanding the etiology and pathogenesis of MMD has been a longstanding challenge in the field of neurosurgery and vascular biology [3, 4]. The disease’s rarity, variable clinical presentation, and complex underlying mechanisms have hindered progress in deciphering its mysteries. To date, MMD is considered a multifactorial disorder with genetic, environmental, and immunological components contributing to its development and progression [5, 6]. However, the precise interplay of these factors and the molecular basis of the disease remains elusive.

The MOYAOMICS project represents a dedicated effort to unravel the intricate tapestry of MMD’s pathogenesis. This multidisciplinary research initiative aims to comprehensively investigate the disease at the molecular level, leveraging cutting-edge technologies in genomics, transcriptomics, proteomics, metabolomics, and single-cellomics. By doing so, we endeavor to shed light on the genetic predisposition, immune dysregulation, and metabolic perturbations that underlie MMD’s complex pathophysiology.

## Methods

The MOYAOMICS project was initiated at the Beijing Tiantan Hospital, Capital Medical University in 2022. The Ethics Committee of Beijing Tiantan Hospital has reviewed and approved this study (KY2022-051-02). The project is being carried out in full compliance with all applicable national and international guidelines, including adherence to the Declaration of Helsinki.

### Study design and population

The MOYAOMICS project has a prospective and exploratory design. The recruitment phase spans a planned duration of 3 years. This initiative will encompass a broad and varied group of MMD patients, matched in terms of age and gender with healthy controls. Inclusion criteria for patients require a diagnosis of MMD confirmed via digital subtraction angiography (DSA) in accordance with the 2012 Japanese diagnostic criteria. It is essential to emphasize that this study adopts a purely observational approach. As a result, all decisions regarding diagnosis and treatment, including the choice of surgical procedures and pharmacotherapy, are exclusively determined by the collaborating neurosurgeons. These decisions are made through a collaborative process with the patient or their guardian, taking into account the patient's overall clinical condition.

**Fig. 1:**
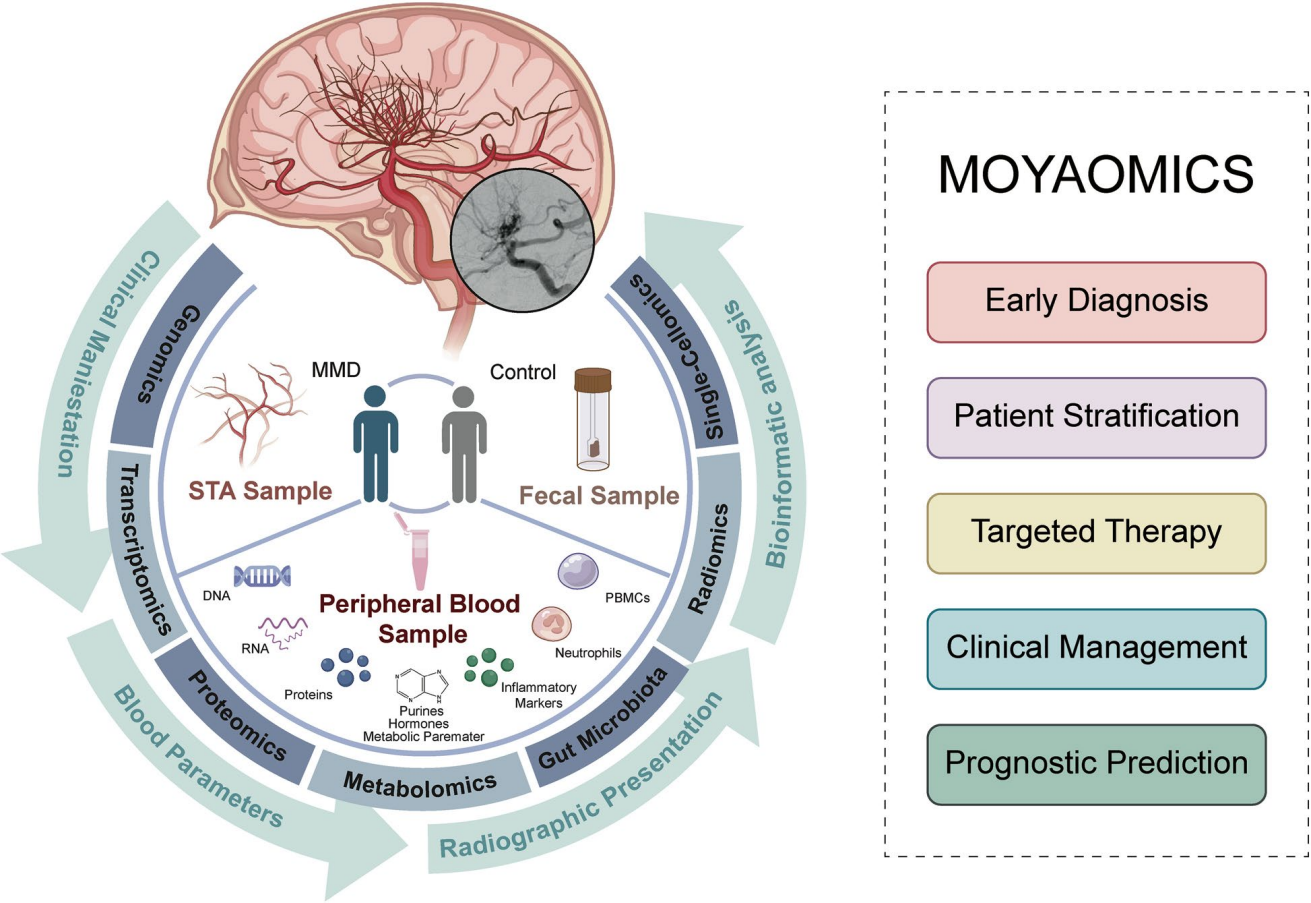
Summary of the major components of the MOYAOMICS

In the study, participants underwent peripheral blood sample collection in the morning, following a minimum 12-h fasting period. A comprehensive overview of the study design is presented in Fig. 1. Peripheral blood samples, totaling 20 ml, were obtained. These samples included ethylenediamine tetra-acetic acid (EDTA) and PaxGene Blood RNA Tubes (Qiagen) for subsequent DNA and epigenetic analysis, such as methylation patterns. Furthermore, we collected a comprehensive array of biological samples, including RNA samples, encompassing messenger RNA (mRNA) and micro-RNA (miRNA). We also obtained neuron-derived exosomes, as well as purines, hormones, proteins, electrolytes, metabolic parameters, and inflammatory markers. Additionally, we collected peripheral blood mononuclear cells (PBMCs) and neutrophils. In individuals diagnosed with either MMD or an internal carotid artery aneurysm, we obtained a small tissue sample from the superficial temporal artery (STA) during the procedure.

Each participant also provided a fresh fecal sample between 06:30 and 08:30 am. These specimens were swiftly transported to our laboratory in well-insulated containers with gel packs, ensuring their maintenance at a temperature of 4 °C throughout the shipping process. Upon arrival at the laboratory, each fecal sample was meticulously divided into five equal portions, each consisting of 200 mg, and immediately preserved at a temperature of – 80 °C. For both healthy controls and MMD patients, samples were preserved at − 80 °C within 6 h and 2 h after collection, respectively. Any samples that had been left at room temperature for a duration exceeding 2 h were subject to disposal.

## Sample size calculation

The calculation for sample size is based on the primary outcome measure, which is the identification of blood-based biomarkers associated with MMD. We aim to detect significant differences in molecular profiles between MMD patients and healthy controls. Based on preliminary data and literature, we estimate that the sample size for each omics is approximately 60 cases of MMD patients and 60 controls. In total, 600 cases of MMD patients are required. This sample size calculation is based on the assumption of a two-sample *t*-test, with a significance level (α) of 0.05 and a desired power (1-β) of 0.80. These calculations have been adjusted for potential dropout rates and the potential presence of confounding factors.

### Inclusion criteria

#### MMD patients

Individuals with a confirmed diagnosis of MMD based on neuroimaging and clinical criteria. Patients with MMD diagnosed by DSA; patients or guardians who agreed to join the study; patients who can complete the relevant examinations involved in this study.

#### Healthy controls

Within this group, we have age- and sex-matched individuals who lack a history of cerebrovascular disease or other significant chronic illnesses. These individuals have been included in the study as a comparison group and have undergone physical examinations at the hospital during the same time frame.

### Outcomes assessment

The primary outcome: analysis of multiomics data between MMD patients and the control group. Collect STA tissue specimens and peripheral blood specimens for genomics, transcriptomics, proteomics, and metabolomics and other multiple omics sequencing analysis, so as to find the relationship between these molecular omics and phenotypes, explore MMD mechanism, including driver genes, activation of signal pathways, etc., and screen sensitive drugs based on potential targets. Use multiomics data to establish an MMD big data analysis platform, including sensitive target prediction, related gene prediction, survival analysis, and so on.

Secondary outcomes: (1) incidence of new ischemic or hemorrhagic stroke [Time Frame: from the baseline to 12 months]. Stroke is diagnosed by symptoms of neurologic deficit or head CT and MRI. (2) incidence of transient ischemic attack [Time Frame: from the baseline to 12 months]. (3) The score of Modified Rankin scale score [Time Frame: change from baseline at 12 months]. (4) Incidence of postoperative ischemic or hemorrhagic stroke [Time Frame: 2 weeks after surgery].

### Data collection

Subject data were meticulously collected by skilled research coordinators through a combination of questionnaires and thorough independent chart reviews. This encompassed a wide range of variables, including demographic information: age, sex, ethnicity, birthplace, residence, and education. Cardiovascular measurements: heart rate, systolic blood pressure, diastolic blood pressure, body mass index. Medical history: hypertension, diabetes, hyperlipidemia, cigarette smoking, alcohol consumption,

### Fasting blood parameters

White blood cell parameters: white blood cell count, lymphocyte count, monocyte count, neutrophil count, eosinophil count, basophil count, lymphocytes, monocytes, granulocytes, percentage of eosinophils, percentage of basophils. Red blood cell parameters: red blood cell count, hemoglobin concentration, hematocrit, mean corpuscular volume, mean corpuscular hemoglobin, mean corpuscular hemoglobin concentration, red cell distribution width, red cell distribution width-coefficient of variation. Platelet parameters: platelet count, platelet distribution width, mean platelet volume, platelet-large cell ratio, platelet crit. Coagulation parameters: fibrin degradation products, D-dimer, prothrombin time, prothrombin time-international normalized ratio, activated partial thromboplastin time, fibrinogen, thrombin time.

Liver function parameters: alanine aminotransferase, aspartate aminotransferase, total protein, albumin, alkaline phosphatase, gamma-glutamyl transferase, total bilirubin, direct bilirubin, cholinesterase, total bile acids. Metabolic parameters: lactate dehydrogenase, creatine kinase, hydroxybutyrate dehydrogenase, glucose, blood urea nitrogen, creatinine, carbon dioxide, uric acid. Mineral and lipid parameters: calcium, phosphorus, triglycerides, total cholesterol, high-density lipoprotein, low-density lipoprotein, apolipoprotein A1, apolipoprotein B. Electrolytes: sodium, potassium, chloride.

Other parameters: homocysteine, globulin, albumin/globulin ratio, indirect bilirubin, estimated glomerular filtration rate.

### Clinical manifestations

This encompassed a range of conditions such as infarction, hemorrhage, transient ischemic attack (TIA), frequent TIAs (occurring ≥ 2 times per month), headaches, and seizures.

### Radiographic presentations at diagnosis

This included critical information about combined aneurysms, Suzuki stage, posterior cerebral artery involvement, and periventricular anastomosis. Notably, this involved the assessment of specific arteries like the lenticulostriate artery, thalamotuberal artery, thalamoperforating artery, anterior choroidal artery, and posterior choroidal artery.

To ensure impartial and rigorous evaluation, radiographic presentations were evaluated independently by two neurosurgeons who conducted blind assessments. In cases where there were differences in interpretation, a third reader was engaged to reevaluate the radiologic presentations. This meticulous and comprehensive data collection approach is instrumental in conducting a comprehensive analysis of the relationship between cardiovascular health, neurological conditions, and associated risk factors.

### Genomics

Extensive genetic investigations, including genome-wide linkage analyses, exome sequencing, association studies, and candidate gene analyses, have uncovered a number of susceptibility genes that are linked to MMD [7–11]. These genes encompass RNF213, MTHFR, TCN2, HDAC9, CBL, and DIAPH1. A prominent genetic factor linked to MMD is the RNF213 p.R4810K variant, which exhibits a high prevalence among Japanese and Korean populations [12, 13]. Nevertheless, it's essential to note that the occurrence of the p.R4810K variant is notably lower in China compared to other East Asian countries [14, 15]. Moreover, only 1 out of 150 carriers of the p.R4810K variant develops MMD, and a significant majority of individuals with this mutated gene have inherited it from an unaffected parent [16]. Consequently, a substantial portion of MMD cases remains genetically undefined, particularly among the Chinese population.

In the genomics segment of our study, we will delve not only into the well-known RNF213 p.R4810K and MTHFR C677T variants but also conduct a comprehensive analysis of the entire genome and exome of MMD patients. Our objective is to identify genetic variations that could potentially contribute to susceptibility to the disease. By harnessing cutting-edge sequencing techniques, we aim to detect single nucleotide polymorphisms, copy number variations, and other genomic alterations linked to MMD. These genetic insights may illuminate the hereditary factors influencing the development of MMD and offer valuable leads for future targeted therapies.

### Transcriptomics

Previous research has provided valuable insights into the distinct patterns of exosomal RNA expression that are associated with the pathogenesis of MMD [17]. Specifically, the upregulation of IPO11 and PRMT1 circRNAs has been linked to the promotion of angiogenesis in MMD, while the underexpression of CACNA1F circRNA is associated with vascular occlusion. In the context of long noncoding RNA profiling within intracranial arteries of MMD patients [18], differentially expressed lncRNAs have hinted at their involvement in various biological processes, including the antibacterial humoral response, T cell receptor signaling pathway, the facilitation of cytokine production, and blood vessel morphogenesis branching.

Moreover, Wang et al. conducted an integrated analysis of lncRNA-mRNA coexpression networks, revealing associations with key biological pathways, including those related to inflammatory responses, Toll-like signaling, interactions between cytokines and their receptors, and the MAPK signaling cascades [19]. Similarly, Gu et al. conducted an investigation into perturbed networks of lncRNAs and competing endogenous RNAs in MMD. Their findings highlighted that the differentially expressed mRNAs within this network were intricately associated with immune responses and inflammatory processes [20]. These processes encompassed activities like the activation of the immune system, the gathering of T cells, the stimulation of T cell activity, the accumulation of lymphocytes, and the initiation of lymphocyte activation.

Transcriptomic analysis will focus on examining the gene expression profiles in the peripheral blood of MMD patients and healthy controls. Through RNA sequencing, we aim to identify differentially expressed genes, enriched pathways, and dysregulated molecular networks that may be pivotal in MMD pathogenesis. This transcriptomic exploration will offer valuable information about the dynamic molecular changes occurring in MMD patients’ blood, potentially pointing toward biomarkers and therapeutic targets.

## Proteomics

Proteomics research has uncovered significant insights into MMD and its underlying pathogenesis. The upregulation of haptoglobin and A1BG in MMD patients' cerebrospinal fluid suggests potential roles in inflammation and angiogenesis, making them promising novel biomarkers for further investigation [21]. Moreover, serum proteomics has revealed dysregulated lipid metabolism in both ischemic and hemorrhagic MMD patients, emphasizing its significance in vascular changes [22].In addition, the characterization of proteins in Moyamoya angiopathy (MMA) between the brain surface and dura mater has identified Filamin A as a potential key player, offering initial insights into MMA’s unique intracranial vasculopathy [23]. Investigation into RNF213’s role in MMD and antimicrobial activity has highlighted changes in proteins like MVP, CYR61, and DDAH1 upon RNF213 knockdown, suggesting potential connections between RNF213, immune responses, and MMD development, possibly involving nitric oxide production in combating Listeria infection [24].

Furthermore, a separate study used quantitative proteomics to analyze serum-derived exosomes from ischemic and hemorrhagic MMD patients [25], uncovering dysregulated proteins associated with cell growth, actin dynamics, and immunity. Treating mouse brain vascular endothelial cells with MMD exosomes enhanced cell proliferation, while proteomic analysis indicated mitochondrial dysfunction in response to hemorrhagic MMD exosomes. These findings provide fresh insights into the potential molecular mechanisms underlying MMD pathogenesis, offering new opportunities for therapeutic exploration.

Moving forward, our proteomic investigations will delve deeper into the protein signatures present in the blood plasma or serum of MMD patients. Leveraging mass spectrometry-based approaches alongside traditional ELISA methods, we aim to identify proteins with differential abundance or post-translational modifications in MMD. This multifaceted approach will enhance our understanding of the disease's underlying mechanisms and signaling pathways. Additionally, it will provide valuable insights into inflammatory processes and protein-protein interactions contributing to MMD pathophysiology. Ultimately, these findings will not only advance disease research and diagnosis but also aid in exploring patient outcomes and prognosis in MMD.

### Metabolomics

Metabolomics, an invaluable tool for deciphering metabolic patterns and disease-specific markers, has been underutilized in adult MMD patients. In a prior study, hydrogen-1 nuclear magnetic resonance spectroscopy was harnessed to analyze cerebrospinal fluid (CSF) metabolites in both bilateral MMD (B-MMD) and unilateral MMD (U-MMD), juxtaposing them with cases of atherosclerotic cerebrovascular disease (ACVD) [26]. The results illuminated specific metabolic biomarkers that are intricately linked with MMD in adults, notably the elevation of glutamine levels distinguishing both B-MMD and U-MMD from ACVD. This metabolomics approach holds tremendous promise for aiding MMD diagnosis and deepening our comprehension of its underlying pathogenesis. A preceding study delved into the plausible role of lipid deregulation in MMD by scrutinizing angiogenic, vasculogenic, and inflammatory proteins alongside lipid profiles in MMD patients and controls (comprising healthy donors and individuals with atherosclerotic cerebrovascular disease) [27]. The findings cast a spotlight on significant disparities in plasma lipid profiles, notably the depletion of glycosphingolipids, hinting at cerebral cellular recruitment. These lipid signatures could play a pivotal role in MMD pathogenesis, offering insights into the intricacies of this disorder.

In a distinct study centering on amino acids, which are essential metabolites crucial for activating the body and brain, researchers analyzed the changes in serum amino acids in MMD patients [28]. Through quantitative scrutiny of serum amino acid metabolic profiles in MMD patients and healthy controls, striking differences came to the fore in the levels of 12 amino acids. Four amino acids—L-methionine, L-glutamic acid, β-alanine, and o-phosphoserine—emerged with notable sensitivity and specificity as potential biomarkers. They present a promising diagnostic tool for early MMD detection and contribute to our comprehension of the underlying pathogenesis of the disease.

Furthermore, our research extends to encompass comprehensive metabolomic analysis, enlisting the examination of small molecule metabolites in both MMD patients and control groups. Leveraging a blend of untargeted and targeted metabolomic approaches, primarily through mass spectrometry, we endeavor to unearth metabolic signatures indicative of MMD presence and progression. This all-encompassing approach not only unveils disrupted metabolic pathways but also harbors the potential to identify invaluable metabolite biomarkers for diagnostic and therapeutic applications. Furthermore, our quest to establish correlations with patient prognoses enables the exploration of metabolites tethered to disease progression, potentially serving as prognostic indicators and furnishing deeper insights into MMD pathophysiology.

### Gut microbiota

The complex interconnection between the gastrointestinal system and the brain, commonly referred to as the gut-brain axis [29–31], reveals a remarkable bidirectional communication. This field of study originated from research involving germ-free animals, particularly germ-free mice, where a decrease in the expression of tight junction proteins was observed. This phenomenon compromised the integrity of both the gut epithelial barrier and the blood-brain barrier, resulting in increased permeability. Intriguingly, a noteworthy discovery emerged when fecal transplantation from specific-pathogen-free mice was employed, successfully reversing the changes in the blood-brain barrier [32]. A wealth of compelling evidence suggests a strong link between alterations in the gut microbiota and a spectrum of central nervous system diseases. These conditions encompass a range of neurological and neuropsychiatric disorders, including but not restricted to Parkinson's disease, Alzheimer's disease, stroke, multiple sclerosis, bipolar disorder, and anorexia nervosa, with ongoing exploration of these intricate relationships [33, 34].

While the precise mechanisms underlying the interaction of the gut-brain axis remain incompletely understood, both animal experiments and clinical studies have illuminated the capacity of the gut microbiota to influence brain behavior and cognitive development. These effects are mediated through diverse pathways, encompassing the nervous system, endocrine system, immune inflammation, and the metabolites orchestrated by the gut microbiota [30, 35]. This collective body of evidence underscores the pivotal role played by the gut microbiota in the regulation of the host's physiological and psychological well-being. The research in this field holds the promise of providing novel insights and innovative approaches for the treatment and prevention of neurological diseases. As investigations continue, they are poised to unveil the intricate mechanisms that govern the interaction between the gut microbiota and the brain, ultimately yielding a deeper understanding with profound implications for future research and clinical applications.

Turning our attention to MMD, one study delved into the intriguing relationship between gut microbiota and this condition. Researchers meticulously examined fecal samples collected from MMD patients, individuals with non-Moyamoya intracranial large artery disease, and control subjects [36]. While the analysis did not reveal significant differences in alpha and beta diversity between MMD patients and controls, it unveiled distinct variations in the relative abundance of specific microbial species. Particularly noteworthy was the increased presence of Ruminococcus gnavus and the decreased presence of Roseburia inulinivorans, both of which were associated with a higher risk of MMD. These findings suggest a potential connection between gut microbiota composition and the pathogenesis of MMD.

In another study focused on MMD, the spotlight turned to brain-gut peptides and their intricate interplay with inflammation. Researchers analyzed serum and CSF samples from MMD patients and healthy individuals, with a specific emphasis on brain-gut peptides (VIP, CCK, SST, SP) and proinflammatory cytokines (IL-1β, TNF-α, IL-12) [37]. Results illuminated significant differences in these biomolecules between MMD patients and their healthy counterparts. Notably, MMD patients exhibited lower levels of VIP, CCK, and SST, while concurrently displaying higher levels of proinflammatory cytokines. Further analysis using multiple logistic regression identified reduced VIP, CCK, and SST levels as independent predictors of MMD occurrence. Intriguingly, negative correlations emerged between these brain-gut peptides and proinflammatory cytokines in both serum and CSF, suggesting a close association between decreased levels of VIP, CCK, and SST and the pathogenesis of MMD. These findings hold potential clinical significance as they offer the prospect of employing these brain-gut peptides, in conjunction with proinflammatory cytokines, as biomarkers for MMD diagnosis and treatment monitoring.

In summary, the investigation into gut microbiota and brain-gut peptides represents a fascinating frontier in the study of MMD. By exploring the composition of gut microorganisms and the roles of specific peptides, these studies aim to unravel the intricate mechanisms underpinning MMD's development. Understanding the influence of gut microbiota and brain-gut peptides on MMD may open up new avenues for therapeutic strategies and dietary interventions tailored to enhance the well-being of MMD patients.

### Radiomics

Radiomics is a specialized field that focuses on extracting quantitative data from medical imaging, such as MRI, CT scans, or PET scans. In the context of MMD, radiomics aims to analyze neuroimaging data to identify subtle patterns, textures, and features that may not be apparent to the naked eye. These advanced image analysis techniques can assist in the early detection, characterization, and monitoring of MMD-related brain changes. Radiomics has the potential to offer non-invasive and objective measures for disease assessment and progression, aiding in both research and clinical management of MMD patients.

Kim et al. investigated the application of deep learning algorithms for the differentiation of MMD using plain skull radiograph images [38]. Their deep learning model achieved an accuracy of 84.1% in predicting MMD, with both sensitivity and specificity at 0.84. Additionally, the study shed light on the potential involvement of the viscerocranium in MMD-related skull features, offering promising prospects for precise MMD diagnosis via deep learning. In a separate study, deep machine learning technology was employed to differentiate between individuals afflicted with MMD and those with atherosclerotic disease, as well as healthy control subjects, utilizing data from magnetic resonance imaging [39]. This approach yielded high accuracies, ranging from 84.8 to 92.8%, across various brain regions, showcasing the potential of AI-based diagnosis in this context. Another research endeavor delved into the utilization of deep learning algorithms to identify MMD and predict instances of hemorrhage [40]. The deep learning models exhibited remarkable accuracy, sensitivity, and specificity in detecting Moyamoya vasculopathy, underscoring their potential for automating diagnosis and promptly recognizing hemorrhagic risk in MMD. Overall, radiomics is an invaluable tool in the study and management of MMD, offering the potential for earlier diagnosis, precise characterization, and objective assessment of disease progression, ultimately improving patient outcomes.

### Single-cellomics

The integration of single-cell RNA sequencing and mass cytometry techniques offers a powerful approach to delve into the intricacies of cellular heterogeneity within peripheral blood samples of MMD patients. This cutting-edge methodology allows for the precise identification and characterization of specific cell populations implicated in MMD pathogenesis. Through the dissection of immune and vascular cell types and the assessment of their molecular profiles, we aim to gain a comprehensive understanding of the immune dysregulation and vascular remodeling that are hallmark features of MMD. Single-cellomics, coupled with mass cytometry, enables us to explore dynamic changes in peripheral immune cells in MMD, shedding light on the intricate interplay of immune responses in the disease.

## Conclusions

The MOYAOMICS project represents a comprehensive effort to uncover the molecular underpinnings of MMD. By integrating multiomics data, we hope to identify key genetic, transcriptomic, proteomic, metabolomic, and single-cellomic signatures associated with MMD pathogenesis. The discovery of blood-based biomarkers could revolutionize early diagnosis, patient stratification, and the development of targeted therapies for this rare and challenging disease. This research has the potential to significantly impact the clinical management and outcomes of MMD patients.

## Data Availability

Not applicable.

## Abbreviations

MMD: Moyamoya disease
MOYAOMICS: Moyamoya Omics Atlas
DSA: Digital subtraction angiography
mRNA: Messenger RNA
miRNA: Micro-RNA
PBMCs: Peripheral blood mononuclear cells
STA: Superficial temporal artery
TIA: Transient ischemic attack
B-MMD: Bilateral MMD
U-MMD: Unilateral MMD
ACVD: Atherosclerotic cerebrovascular disease
MMA: Moyamoya angiopathy
